# Processing Chinese Relative Clauses: Evidence for the Subject-Relative Advantage

**DOI:** 10.1371/journal.pone.0077006

**Published:** 2013-10-02

**Authors:** Shravan Vasishth, Zhong Chen, Qiang Li, Gueilan Guo

**Affiliations:** 1 Linguistics, University of Potsdam, Potsdam, Brandenburg, Germany; 2 School of Mathematics and Statistics, University of Sheffield, Sheffield, South Yorkshire, United Kingdom; 3 Linguistics, Cornell University, Ithaca, New York, United States of America; 4 Institute for Language and Cognition, Dalian University of Technology, Dalian, Liaoning Province, China; 5 Linguistics, University of Potsdam, Potsdam, Brandenburg, Germany; University of Leicester, United Kingdom

## Abstract

A general fact about language is that subject relative clauses are easier to process than object relative clauses. Recently, several self-paced reading studies have presented surprising evidence that object relatives in Chinese are *easier* to process than subject relatives. We carried out three self-paced reading experiments that attempted to replicate these results. Two of our three studies found a subject-relative preference, and the third study found an object-relative advantage. Using a random effects bayesian meta-analysis of fifteen studies (including our own), we show that the overall current evidence for the subject-relative advantage is quite strong (approximate posterior probability of a subject-relative advantage given the data: 78–80%). We argue that retrieval/integration based accounts would have difficulty explaining all three experimental results. These findings are important because they narrow the theoretical space by limiting the role of an important class of explanation—retrieval/integration cost—at least for relative clause processing in Chinese.

## Introduction

One of the central concerns of sentence comprehension research has been to identify universal, that is, cross-linguistically applicable, constraints that determine online parsing difficulty. The underlying assumption is that the human sentence comprehension mechanism is subject to certain universal principles which should apply regardless of the language in question. However, it is obvious that, to some extent or another, languages differ. Nevertheless, the possible existence of a core set of universal parsing constraints is a very attractive proposition and researchers in psycholinguistics have pursued it with vigor, often with remarkable success.

An example of a cross-linguistically consistent result concerns relative clause (RC) processing. A robust finding in the literature is that, at least as far as sentences with animate, definite, and full noun phrases are concerned, subject relatives (SRs) (example: *The man who talked to the woman was a doctor*.) are easier to process than object relatives (ORs) (example: *The man who the woman talked to was a doctor*). A subject relative is a sentence where a noun (here, *man*) is modified by a relative clause (here, *who*… *woman*), and the modified noun is the grammatical subject of the sentence. In an object relative, the noun modified by the relative clause is the grammatical object of the relative clause.

English shows a subject-relative advantage, as demonstrated by a number of studies involving different methods such as: lexical decision [1]; self-paced reading [2], [3]; eyetracking [4]; event-related potentials [5]; functional magnetic resonance imaging [6]–[8]; and positron emission tomography [9].

The subject-relative preference is also observed in languages other than English. Some examples are: Dutch [10], [11], French [12], German [13], [14], Japanese [15], and Korean [16], [17]. The subject-preference extends to cross-linguistic second-language acquisition studies as well [18], [19].

### Chinese and the cross-linguistic subject relative advantage

This universal processing pattern of RCs has inspired many explanations (see [20] for a comprehensive summary). We focus on two alternative explanations which make opposing predictions for Chinese relative clauses (which is the focus of the present paper).

1. Frequencies of Relative-Clause Types [21]. This account claims that since SRs tend to occur more frequently than ORs parsing an SR should be easier. Corpus evidence shows that, cross-linguistically, SRs are indeed more common than ORs. For example, in the Brown corpus of the English Penn Treebank (http://www.cis.upenn.edu/~treebank/) the frequency distribution of SRs versus ORs is 86% and 13% [22]; in the German NEGRA corpus [23], it is 74% and 26% [24]; and in the Chinese Treebank, 57.5% and 42.5% [20]. For all languages where this pattern holds, an SR advantage is predicted.
2. Working memory accounts: As the review in [25] summarizes, there are two main classes of working memory account: (i) Storage-based theories, which argue that object relatives are harder to process because a larger number of predictions have to be maintained in memory compared to subject relatives; and (ii) Integration/retrieval accounts, which predict that object relatives are harder to process because the distance between a head and its dependent is greater in object relatives (these two accounts are discussed in more detail below).

These two alternative accounts of relative clause processing are interesting in the context of Chinese, because this language presents a puzzling irregularity in the universal subject preference. (As an aside, we note that Basque also represents an exception to the universal subject-relative preference, but the Basque facts apparently have to do with the presence of ergativity in that language [26]; although there are at least two ergative languages with a subject-relative advantage [27], [28].) Hsiao and Gibson [20] found that Chinese ORs appear to be easier to process compared to SRs. This Chinese result is particularly interesting because it derives the Chinese facts from a universally applicable working memory cost metric (the Dependency Locality Theory [29]) that includes storage and integration cost (discussed below). The result is also interesting because it runs counter to subsequent experimental findings involving Chinese RCs, which found a subject preference [30]–[34]. Note, however, that there also exists evidence consistent with Hsiao and Gibson's finding (e.g., [25], [35]; also see Table 1).

**Table 1:** Summary of previous reading studies on Chinese relative clauses.

	Study	Y	V	n	method	location
1	[25]	−123.20	46.84	36	SPR	Taiwan
2	[77] expt 1	−100.00	30.00	48	SPR	Taiwan
3	[39] expt 1	−70.00	42.00	32	GMaze	USA
4	[77] expt 2	−30.00	42.35	40	SPR	Taiwan
5	[39] expt 2	6.19	19.90	24	LMaze	Shanghai
6	[20]	50.00	25.00	35	SPR	USA
7	[40]	50.00	42.35	48	SPR	Shanghai
8	[41]	50.00	23.00	40	SPR	Shanghai
9	[73]	55.62	65.14	49	SPR	Nanjing
10	[42]	75.00	35.50	39	SPR	Beijing
11	[43]	81.92	36.25	49	ET	Taiwan
12	[30]	100.00	80.00	48	SPR	Taiwan

We show the estimated coefficient and estimated standard error from previous studies that we had access to; the estimated coefficients are sorted in increasing order; the sample size (number of participants); and the method used. SPR means self-paced reading, ET means eyetracking, and the Maze tasks in Qiao et al are described in their paper. A negative coefficient means an object relative advantage, and a positive coefficient a subject relative advantage. See main text for details.

In order to understand the theoretical implications of the original Hsiao and Gibson results on theories of RC processing, it is necessary to examine their findings and claims in detail. Using self-paced reading [36], Hsiao and Gibson argued that in Chinese, a language with subject-verb-object word order and with prenominal RCs, reading time (RT) in SRs was slower than in ORs in several theoretically interesting regions of the sentences.

They relied on single and double-center embedded relative clauses as shown in (1); the word *de* functions in this context like the relativizer *who* in English RCs (but it is not a relative pronoun; see corpus study discussed below). The reading experiment was preceded by a plausibility norming task, in which participants were “asked to judge the naturalness in the real world of the events described in the sentences, that is, how likely they were to occur” [20], [10]. Only those sentences that were matched for event-plausibility were used in the experiment.

(1) a. Single-embedded SR

**[**GAP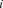 yaoqing fuhao de**]** guanyuan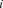 xinhuaibugui

invite tycoon DE official have bad intentions

‘The official who invited the tycoon has bad intentions.’

- b. Single-embedded OR

**[**fuhao yaoqing GAP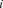 de**]** guanyuan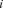 xinhuaibugui

tycoon invite DE official have bad intentions

‘The official who the tycoon invited has bad intentions.’

- c. Double-embedded SR

**[**GAP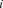 yaoqing **[** GAP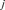 goujie faguan de**]** fuhao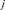 de**]** guanyuan
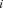


xinhuaibugui

invite conspire judge DE tycoon DE official have bad intentions

‘The official who invited the tycoon who conspired with the judge has bad intentions.’

- d. Double-embedded OR

**[[**fuhao yaoqing GAP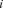 de**]** faguan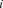 goujie GAP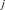 de**]** guanyuan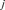 xinhuaibugui

tycoon invite DE judge conspire DE official have bad intentions

‘The official who the judge who the tycoon invited conspired with has bad intentions.’

The main results of their experiment were as follows. In single embeddings (1a,b), the first two words in ORs (1b) were processed faster than the first two words in SRs (1a). No significant differences were found at *de* or the following region (i.e., head noun) in single embeddings. In double embeddings (1c,d), no significant differences were found at the first two words, but ORs (1d) were processed faster than SRs (1c) in the region containing the third and fourth words combined (the object NP and *de* in SRs, and *de* and subject NP in ORs). The same pattern was found for the RTs in the fifth word, and the sixth word.

The object preference in Chinese RCs that Hsiao and Gibson reported dramatically shrinks the space of theoretical explanations. Of the two classes of theories mentioned above, only the working-memory explanation can account for the object preference. The Chinese results appear to rule out the frequency-based account, and any other account predicting a subject-relative advantage, quite decisively. Hsiao and Gibson explain the results in terms of Dependency Locality Theory or DLT [29], [37]. The DLT is a remarkably elegant and simple theory with two processing cost metrics. One metric is integration cost, which asserts that assembling a dependency is a function of the linear distance between the co-dependents (e.g., gaps and displaced nouns, or arguments and verbs), where distance is defined as the number of intervening discourse referents. The other metric is storage cost, according to which memory resources are consumed as a function of the number of upcoming heads predicted.

The integration cost metric explains the Chinese subject-object asymmetry as follows. Simplifying somewhat and focusing only on single embeddings, compared to the OR's head noun (1b), the SR's head noun *guanyuan* in (1a) is more distant (with an intervening discourse referent *fuhao*) from the gap it is coindexed with than the head noun in the OR (1b) (see [20], [7]). Integration cost at the head noun is therefore higher in the SR. Here, we follow [20] in assuming that the location of the gap in the object relative clause is to the left of the relativizer *de*. This is possibly a questionable assumption [16], but has no implications for the predictions of the integration cost metric.

The storage cost metric can also explain the RC asymmetry. Storage cost at a given word is defined as the number of predicted heads [20], [29]. Consider again the SR (1a), which begins with the verb *yaoqing*. Hsiao and Gibson assume that a verb-initial Chinese sentence presented out of context (as opposed to a discourse context that licenses a null subject such as a *pro*) should lead the reader to expect an RC structure, leading to three predicted heads: the object of the verb in the RC, the relativizer *de* and the predicate of the matrix clause. By contrast, in ORs (1b) the initial word is a noun, which leads the reader to predict only one head, an upcoming verb because this noun could be the subject of a matrix clause. Thus, at the first word, storage cost predicts that SRs are harder than ORs. This difference should continue right up to the relativizer *de* in single embeddings. As [25] point out, at the relativizer, a reanalysis should occur in the object relative because at this point the reader realizes for the first time that the sentence is a relative clause. Thus, from the relativizer onwards, an SR advantage is predicted. A relevant point to note is that if preceding context leads the reader to believe that both the SR and OR are relative clauses (i.e., if there is no possibility of a garden-path), then only the predictions of the integration cost metric are of interest. This fact becomes relevant when discussing Gibson and Wu's experiment [25], which provided preceding context to eliminate such garden-pathing.

The Hsiao and Gibson experimental materials and design have come under criticism from Lin and Bever [30], who argue that the results for both the single embeddings and double embeddings were confounded by other factors. Recall that in the single embeddings, ORs were read faster in the pre-relativizer region; Lin and Bever argue that this may be simply because in SRs (as Hsiao and Gibson also assume) an empty subject must be posited and this may be computationally costly. In double embeddings, Lin and Bever argue, the increased processing time at the second and third words combined, and at the fourth and fifth words separately, could be due to the differing distance between the gaps and fillers (in effect, the locality) in the RCs, rather than differing storage costs. Lin and Bever also point out that Chinese SRs have nested dependencies in doubly-embedded RCs, whereas object double embeddings have presumably easier-to-process serial dependencies. This difference is presented by them as a confound for the Hsiao and Gibson findings. However, the nested-versus-serial dependency confound can be recast in terms of integration cost, so it is not clear whether this is really a confound or a different explanation for the results, one relying on integration cost rather than storage cost.

Lin and Bever also argue that some of the verbs used in the Hsiao and Gibson experiment were ambiguous in the type of arguments they took, which could confound the results (presumably due to differing storage costs in ambiguous verbs). Lin and Bever then demonstrate through two experiments that Chinese SRs are easier than ORs, as in the case of virtually every other language examined.

Lin and Bever's work was in turn criticized by Gibson and Wu [25] on the grounds that they only found the SR advantage in object-modifying relative clauses, not in subject-modifying relative clauses. Gibson and Wu argue that in object-modifying relatives, a temporary garden path occurs in the object-relative because the sentence begins with a noun-verb-noun structure that leads the reader to expect a main verb construction. Such a garden-path does not occur in subject relatives where the object is modified.

In order to demonstrate the subject-relative advantage in subject-modifying cases, Gibson and Wu [25] carried out an experiment using subject-modifying relative clauses which were preceded by an appropriate context such the target sentence was assumed by the reader to be a relative clause. They found the following:

1. No differences were found between the subject and object relative at the first two words, although the SR condition was numerically slower.
2. At the relativizer, they found significantly slower RTs in the SR than the OR, but this difference was not reliable in the by-items analysis.
3. At the head noun, the SR was read significantly slower than the OR.

Thus, compared to Hsiao and Gibson's study [20], the major difference in the Gibson and Wu study is the significant effect at the head noun: in the earlier study they found no effect in single embeddings (more precisely, they found a numerical subject-relative advantage that did not reach significance), and in the Gibson and Wu study an OR advantage is seen at the head noun. The Gibson and Wu study therefore presents clear evidence in favor of the integration-based explanation.

### The motivation for attempting a replication of previous studies

Although replication of published work is always desirable in experimental research, we felt it was particularly necessary to attempt a replication of studies regarding Chinese relative clauses. There are two reasons for this. First, unlike the case of comparable structures in English relative clauses, the existing reading studies on Chinese relative clauses show quite a lot of variability; second, we felt that some of the conclusions from previous studies could be problematic. We discuss both these points below.

In Table 1 we summarize the effects found in previous reading studies at the head noun (we limit the discussion to the head noun in this paper because we are primarily addressing the claim in [25] regarding integration costs, which come into play at the head noun). We only look at studies involving singly-embedded subject vs. object relative clauses and animate nouns. For each study, we initially attempted to obtain the raw data from the respective authors; this gave us data from Gibson and Wu's study, and the two experiments conducted by Qiao and colleagues. For the remaining studies, we were either unable to obtain data or did not attempt to obtain it; for these studies we estimated the location and scale parameters from the published results. This was impossible to do in the case of the self-paced reading studies in [38] because no statistical information was provided in the paper regarding scale parameters, so we do not include data from that paper.

Some comments are necessary on how we extracted the estimated coefficients and standard errors from the various papers. Qiao et al [39] trimmed 2.5% of their data; as they describe it (p. 7): “Any observation was excluded if it differed by more than 2.5 standard deviation units from the value predicted by the model.” Since the two studies by Gibson and colleagues did not do any trimming, and since we had access to the Qiao et al data, we computed, for both their experiments, the untrimmed mean difference between subject and object relatives and the estimated standard error of this difference (only correct responses were considered in these data, as in Qiao et al's paper). A statistical analysis with untrimmed data (a maximal linear mixed model using a negative reciprocal transform; see below) did not in any case show any evidence that any trimming procedure was needed. In the study by Wu and colleagues that compared subject and object relatives [40] only log reading times are reported; here, we estimated the coefficient from the figure they display. The standard error was estimated as the mean of all the standard errors in other studies. Similarly, Wu's PhD dissertation [41] presents a similar experiment to [40]; it also compares subject and object relatives and reports a subject-relative advantage at the head noun (p. 178), and a by-subjects F-score of 5.525 with degrees of freedom 1,39. This implies a t-value of 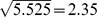. Since Wu's results (Fig. 4.3, p. 177) show approximately a 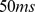 SR advantage, we can estimate the standard error as 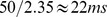 (from the fact that 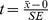), a reasonable estimate given the other experimental data available. In the case of Hsiao and Gibson's data for single embeddings [20], we estimated the effect and standard error from the figure provided in the paper (we ignore the double embedding data for the reasons discussed earlier). Finally, Chen et al [42] report (p. 64): “at word N2 [the head noun], the main effect of sentence type was significant by participants analysis, F1(1,38)  = 4.46, MSE = 105,506, p 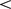 0.05, but not significant by items analysis, F2(1,22) = 0.39, MSE = 29,483, p = 0.54.” Although not remarked upon in the paper, the authors are reporting a *subject* relative advantage here, which directly contradicts the title of their paper, *Chinese subject-relative clauses are more difficult to process than the object-relative clauses*. Finally, we use total reading times at the head noun from the eyetracking study by Jäger and colleagues [43] because these have been argued to approximate self-paced reading results [44]–[47].

Table 1 does not include experiments, such as the one conducted by Wu and colleagues [40], that investigated the animacy status of noun phrases within relative clause type because in these cases there is no direct comparison between SRs and ORs. It also does not include unpublished experiments (which showed a subject relative advantage) for which we could not obtain permission to present the effect size; thus, the table, if it were to fully represent our current state of knowledge of Chinese relative clauses, would have four studies favoring an object relative advantage, and more than eight studies favoring a subject-relative advantage.

For consistency regarding the unit of the dependent measure, we focus only on reading studies or studies that are meant to approximate reading comprehension (e.g., the Maze task of Qiao and colleagues). There is also evidence from event-related potentials studies such as [34] for an object relative advantage; Packard and colleagues found a positivity (P600) at the relativizer region in subject relatives.

The above summary of reading studies shows that previous work has reported a broad spectrum of results, from an object relative advantage of 123 ms to a subject relative advantage of 100 ms. This is in stark contrast to the English relative clause literature, where we do not see such variability for comparable structures. This uncertainty in previously published results makes it particularly important to attempt replications.

A further motivation for attempting a replication is that the Hsiao and Gibson study and the Gibson and Wu study raise several worries. Regarding the Hsiao and Gibson study, as Hsiao discusses in her dissertation [48], the mean age of the participants was somewhat higher than in relative clause studies conducted on university student populations. The mean age was 45 years (p. 60 of [48]). Furthermore, the experiment itself was conducted in different places, and not always in a standard experimental laboratory. Both the dissertation and paper point this out; quoting from the dissertation [48]: “Six [participants] are from MIT (Boston/Cambridge) and the surrounding community. Seven reside in Taiwan, but were attending a wedding in California at the time of the experiment. The other twenty-seven are based in and around Los Angeles. All are native speakers of Mandarin Chinese spoken in Taiwan and use Mandarin Chinese daily (percent of Chinese use: 50–100%).” Although in principle a non-laboratory setting does not necessarily lead to bias (in fact, in some cases there is no choice but to conduct the study outside a laboratory), it is nevertheless worth repeating the experiment in a laboratory setting.

Another worry in this dataset is that the question-response accuracies for single center embeddings are unusually low, at 76% (OR) and 71% (OR) for single embedded relatives. This seems to be the only study on Chinese relative clauses with such low question-response accuracies. It is therefore possible that the participants were not really attending much to the sentences. The questions used in this experiment are not available with the paper or the dissertation, but the paper reports that the questions were not particularly demanding (p. 9): “The comprehension questions for target items questioned the content of the main clause or one of the RCs. For example, two possible questions for sentence (3a) would be “Did the official invite the tycoon?” (no) or “Did the official have bad intentions?” (yes). By contrast, in [25], the accuracies for single center embeddings are 91.2% (both SRs and ORs). In this paper, the questions were about both the target sentence and the preceding context, i.e., if anything, they should have been at least as difficult, if not more difficult, than the original study. Gibson and Wu report that “[t]he comprehension questions asked about either the content of the context clauses or the final clause (containing the RC for the target materials). For instance, the comprehension question for the example in (6) was “Did the car chase take place through light traffic?”, and the answer was “no”. For the 16 target materials, eight asked about the context and eight asked about the RC.” In our own three attempted replications (discussed below), the accuracies are in the range 80–90% for single embeddings.

We also found surprising the fact that in the Hsiao and Gibson study, the question-response accuracy for double embedded object relative is approximately the same as the single embedded object relative; this is very surprising, given that Chinese double embeddings are much more difficult to process. Such a question-response accuracy pattern would be plausible if the comprehension questions were superficial. However, in the paper, it appears that questions were properly counterbalanced to target both the main clause and the relative clauses, as mentioned above. Given these potential issues in the original study, it is worth at least attempting to replicate the experiment results.

The more recent Gibson and Wu results [25] could also be difficult to interpret, but for different reasons. For brevity, we focus only on the results at the head noun since this is the critical region for the integration account. We present a more complete analysis in Table 2.

**Table 2:** Analysis of Gibson and Wu data.

	coef	SE	t-value
VN/NV	−0.01	0.04	−0.37
de	−0.15	0.07	−2.01
head noun	−0.08	0.09	−0.84
head noun+1	0.04	0.08	0.51

Analysis of Gibson and Wu data using negative reciprocal reading times and maximal models. A negative sign on the estimated coefficient is an object relative advantage.

The central finding in that paper is evidence for integration cost at the head noun (they did not find any evidence for storage cost predictions). Gibson generously released the raw data, which allowed us to reanalyze their data following their analysis methodology (they used an F-test on raw reading times; we use a paired t-test, which is identical since t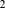 = F)). We were able to replicate their published results using raw reading times (t(36) = 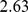, p-value = 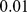, difference between the means 123 ms, 95% CIs 28, 218 ms). As an aside, we note a minor issue in the data: subject 27 has only 7 data points compared to 15 data points for each subject. We kept this subject in the analysis in order to follow the published result. Perhaps a more conservative statistical analysis would use linear mixed effects models; indeed, a maximal linear mixed effects model [49], with varying intercepts for item and subject, and varying slopes for condition (condition is coded as −0.5 for subject relatives, and 0.5 for object relatives), failed to show an effect at the head noun for raw reading times (estimated coefficient: −119.7 ms, standard error 67.5 ms, t-value: −1.77). This leads to a quite different conclusion than the ANOVA-based analysis: no significant evidence for an object-relative advantage.

**Figure 1:**
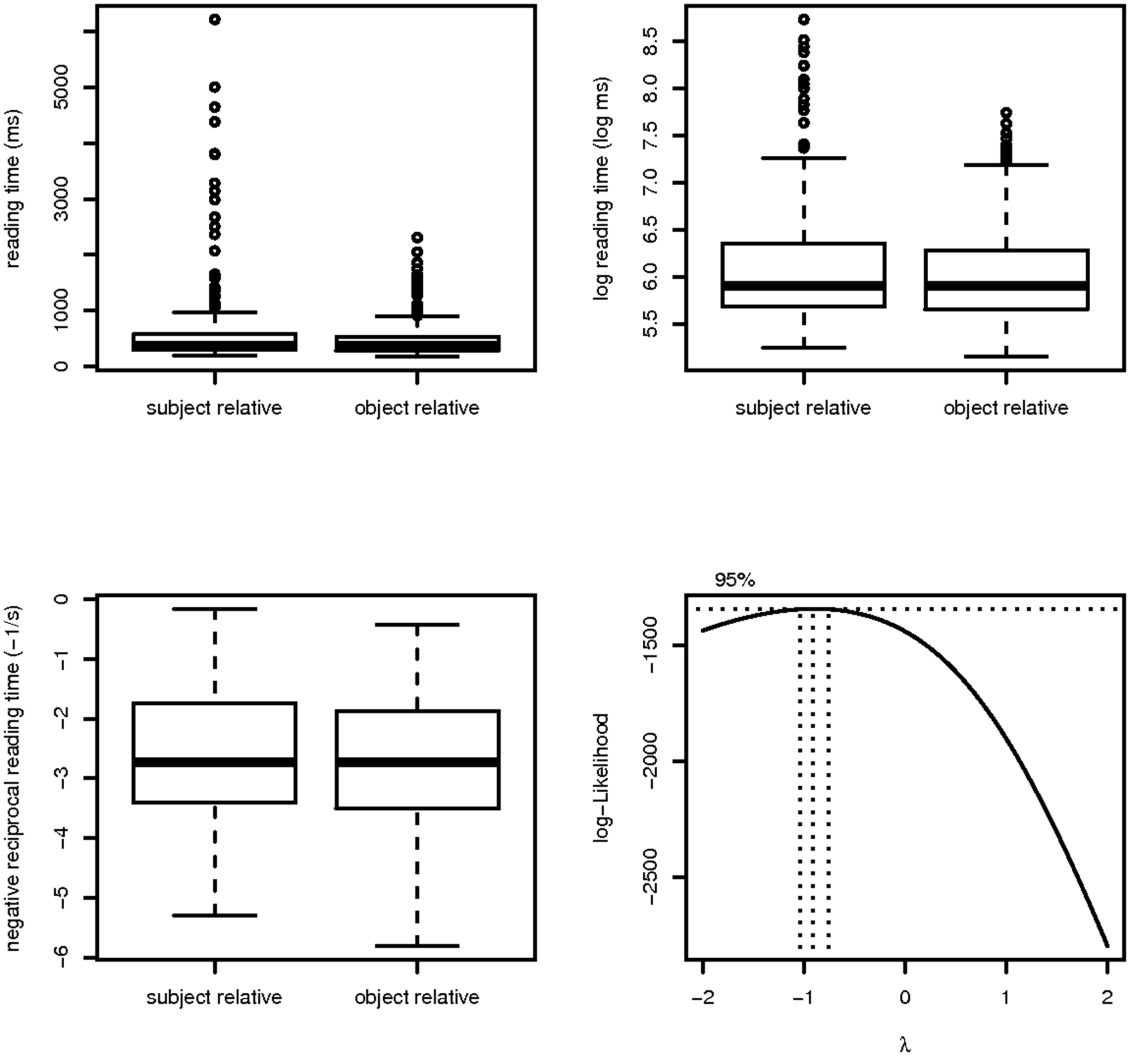
The distributions of the raw, log-transformed, and negative-reciprocal transformed data at the head noun in the Gibson and Wu dataset. The raw reading times were the ones reported in the paper. The bottom-right plot show the results of the Box-Cox procedure; the procedure suggests a reciprocal transform for stabilizing variance.

As shown in Figure 1, an important issue in the Gibson and Wu data is that the distribution of the raw reading times is heavily skewed in the subject relative clause condition only. For brevity we show the results at the head noun, which is the region where a significant difference was found. For a full analysis of all regions, please see the code and data provided with this paper. Indeed, the published subject relative advantage at the head noun in Gibson and Wu's paper is driven entirely by 13 extreme data points (out of 547; i.e., by 2% of the data); these are reading times occurring in the subject relative clause condition that exceed 2,300 ms.

**Figure 2:**
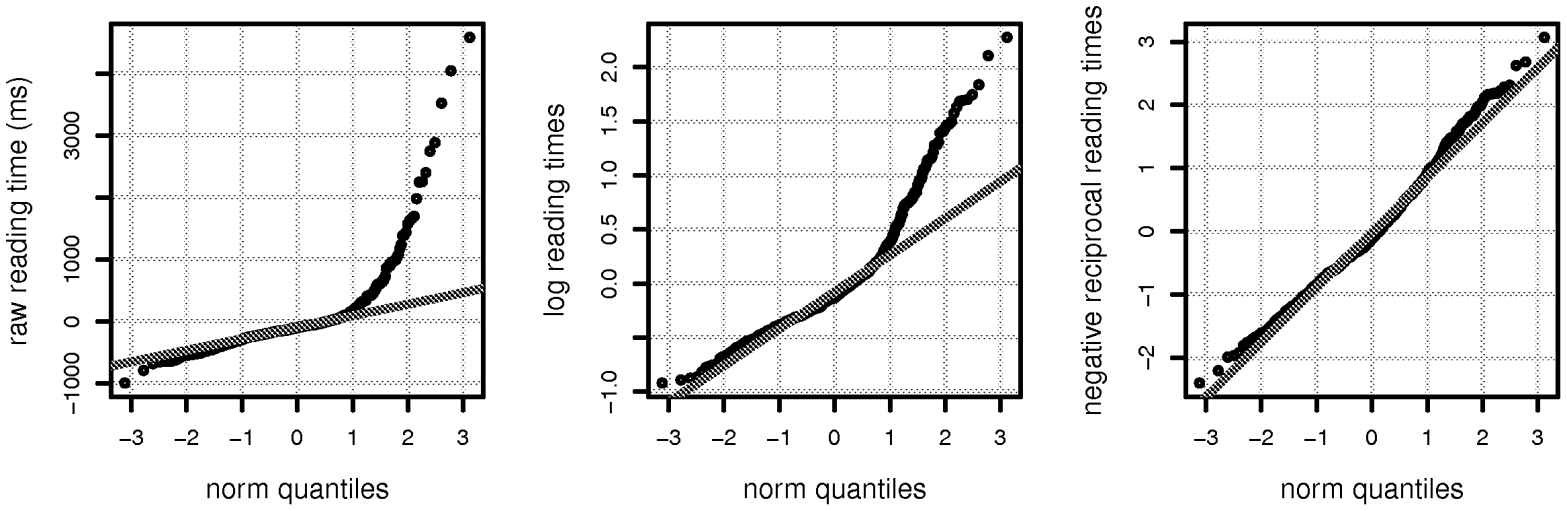
The distributions of residuals from maximal linear mixed effects models for the raw, log-transformed, and negative-reciprocal transformed data at the head noun in the Gibson and Wu dataset.

Figure 2 shows that an important distributional assumption in the statistical model is not satisfied for raw reading times. We used the Box-Cox procedure [50] to determine that a reciprocal would be an appropriate transformation for obtaining normally distributed residuals. Once this transformation was carried out, a linear mixed model with varying intercepts and slopes at the head noun showed no effect at all (t = −0.85); compare this to the t-value in the maximal linear mixed model with raw reading times (t = −1.77) and the t-value in the published analysis using a paired t-test on aggregated scores (t = 2.63). A paired t-test on the transformed scores shows similar null results. Since log transformation is a widely accepted general approach to transforming reading time in psychology, we also analyzed the data using log transformation; this also showed no effect (t = −1.5). Thus, we conclude that the Gibson and Wu dataset does not present evidence in favor of the object relative advantage, and there is also no evidence for integration cost at the head noun in this data.

As an aside, we note here that checking model assumptions is generally considered unnecessary in psycholinguistic research; most studies publish analyses on raw reading times, which almost always result in a violation of the normality assumption in residuals. The normality assumption is not checked because it is widely believed that statistical models are robust to violations of normality of residuals. While it is true that parameter estimation does not require distributional assumptions, and that in some cases the normality assumption can be relaxed, for the relatively large datasets used in psycholinguistics, hypothesis testing crucially requires that the normality assumption regarding residuals is satisfied. The belief in the unimportance of model assumptions has been strengthened by statements by leading statisticians, such as Gelman and Hill, who have written [51], [46] that the normality of residuals is the least important aspect of fitting linear models. At least in the psycholinguistics world, “least important” has been misinterpreted to mean “unimportant for all purposes” (in fact, even Gelman recommends transforming reading time data to the log scale; see his blog entry http://andrewgelman.com/2013/08/04/19470/). Estimating the variance of parameters, computing confidence intervals, and doing hypothesis testing depend on distributional assumptions being satisfied; this is one reason why the Box-Cox procedure is used in statistical data analysis. We have encountered the objection to transforming reading time data that we are no longer in the millisecond scale; however, most theories in psycholinguistics (even computationally implemented theories) do not make quantitative predictions about the magnitude of the effect. Rather, the predictions are only about the direction of the effect. In such a situation, the scale of the dependent measure does not play a critical role; the model assumptions do.

The above issues led us to attempt a replication of these studies. We began this investigation by carrying out a corpus count of relative clauses. This study was completed in 2006, as the diploma thesis of Gueilan Guo, “Predictability of Chinese Relative Clauses”; since 2006, several other corpus studies that have been published, among them [40], [41], [52], which also look at animacy interactions in relative clauses. The corpus study was motivated by a desire to better understand the predictions of the structural frequency-based explanation. We follow up this corpus study with self-paced reading experiments that compare SRs and ORs (i.e., (1a) and (1b)). We are particularly interested in the subject-object asymmetry at the head noun and at the words following it; these regions are of central importance to the current controversy about the integration cost as an account of RC processing in Chinese.

Our corpus study serves to confirm the claims about the relative frequencies of subject and object relative clauses in Chinese. This is important as a starting point in order to determine whether the assumptions made about incremental structure building are correct. Then we present the three self-paced reading studies which present evidence consistent with a subject relative advantage. Our findings are consistent with the predictions of structural frequency-based accounts but not with working-memory based accounts.

## Results

### Results of corpus study

A central assumption in the Hsiao and Gibson paper was that seeing a verb-noun sequence sentence-initially would lead the reader to predict a subject relative clause. We carried out a corpus study to investigate what the most probable structure would be when a verb-noun sequence occurs sentence-initially.

We searched a text corpus developed in Taiwan, the Sinica Corpus 3.0. This corpus, which contains 5 million words and is tagged for parts of speech, is a balanced corpus of texts ranging from 1990 to 1998.

The corpus search yielded 639 sentences with the SR pattern *V N* 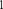 de *N* 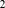 and only 117 with the OR pattern *N* 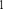 *V de N* 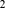. Since the SRs and ORs used in the Hsiao and Gibson experiment only had animate N
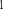
s and N
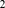
s [20], [17]–[26], we isolated the four sub-types of the putative SR-OR patterns to establish whether constraining the RCs to animate ones yielded any new information. The results are summarized in Table 3.

**Table 3:** Summary of corpus search.

	SR-like structures:		OR-like structures:	
	N 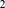 animate	N 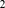 inanimate	N 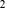 animate	N 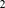 inanimate
N 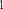 animate	**13**	51	**3**	**42**
N 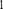 inanimate	**106**	469	1	71

A partition of SR-like and OR-like strings by NP-animacy. The numbers in bold are bona fide RCs. The other patterns are discussed in the text.

These frequency patterns are consistent with Hsiao and Gibson's corpus search, which showed a higher frequency of SRs; there were 119 SRs and 45 ORs. If one restricts the noun phrases to animates only, 13 SRs and 3 ORs were found.

Note, however, that the majority of the RC-like structures are not RCs, they merely have the same sequence of words as an RC. One consequence is that it is no longer obvious how the parser would use frequency information in incremental online processing. We spell out next the frequency-driven decisions of the parser.

First, consider SRs. Sequences like *V N* 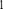 *de N* 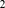 form SRs only in 19% of the cases (106+13 = 119 of the 639 sentences are SRs). The majority (469) had the inanimate *N* 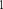 combined with *de* to form a possessive modifier of verb's inanimate object *N* 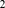 (2), and 51 had the *V N* 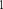 sequence (with an animate N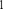) as a possessive modifier of an inanimate N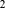 (2b).

(2) a. tisheng [ [ qiye de ] jingzhengli ]

increase company DE competitiveness

‘To increase the company's competitiveness.’

- b. [[guyong yuangong] de] chengben…

hire employee DE cost

‘The cost of hiring an employee …’

Neither of the structures in (2) is an SR and they are much more frequent than SRs. This has two implications for the predictions of the frequency account and the storage cost metric of the DLT.

First, if frequency can arbitrate in incremental parsing decisions, when the first verb is processed in a putative SR, the parsing mechanism would not choose the (gapped) RC structure as the most likely parse. It may well be the case that a non-RC structure is posited. If this is correct, this goes against the storage cost assumption that Hsiao and Gibson [20] present for subject relatives: they assume that a V N sequence beginning a sentence necessarily entails a subject relative construction because of the low probability (i.e., the low frequency) of verb-initial non-relative clauses.

Our study suggests that a non-relative clause could be the more likely prediction. Among V N de N sequences, when the first noun is animate, subject relatives occur with probability 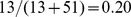 (see Table 3). If the noun in the V N structure is inanimate, a relative clause structure has probability 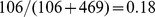, much lower than a non-relative clause structure. Thus, it is difficult to conclude that the storage cost metric predicts greater difficulty for subject relatives due to the V N sequence leading the reader to posit a relative clause.

Second, once the animate first NP is seen in the SR, if a second NP is predicted at all it is more likely to be an inanimate NP in a non-RC structure: animate first NPs tend to be followed by inanimate second NPs (51 instances, which are not RCs) more often than by animate second NPs (13 instances that are RCs). The same situation holds at the relativizer *de*: it is more likely that the continuation is a non-relative.

The above two facts suggest that at the SR head noun, the comprehender would experience a surprise due to the falsified expectations that (i) the head noun is inanimate and (ii) the structure is a non-RC.

Let us consider the situation in ORs next. In ORs, among all the sequences that had the same pattern as RCs, a majority involved inanimate nouns only (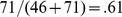), and they were not ORs. An example is (3).

(3) chaye jinkou de shuliang…

tea-leaves import DE quantity

‘The quantity of imported tea-leaves…’

In these structures, the first NP was not the subject of the RC, but rather a compound noun *chaye jinkou*, ‘tea-leaves import’, and the *de* serves as a genitive modifier of the NP *shuliang*, ‘quantity’. The proportion of bona fide ORs was 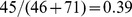, and, as mentioned above, the proportion of cases where both NPs were animates (as in the case of the Hsiao and Gibson's stimuli) was 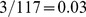.

Here, as in SRs, once the comprehender sees the animate first NP and the following verb, the most favored expectation may not be a relative clause (this is consistent with Gibson and colleagues' assumption). Even when the character *de*, functioning as a relativizer, is seen next, a non-RC structure is a more likely continuation than an RC. At the head noun, surprise should be experienced, due to two distinct factors: (i) there is a greater expectation for an inanimate NP rather than animate, and (ii) the structure is recognized for the first time as an RC.

Thus, in the animate-only structures considered by Hsiao and Gibson, since SRs and ORs are ambiguous between relative and non-relative constructions up to the point when the head noun is processed, both SRs and ORs should experience processing slowdowns at the head noun, and part of this slowdown can be attributed to the unexpected animacy of the head noun (note that [35], [40], [41] have shown the importance of animacy information in the processing of Chinese relative clauses). In particular, the storage cost predictions of Hsiao and Gibson do not seem plausible given the frequency of the different possible structures. We therefore set aside the storage cost metric as the main target for study in the present paper, although we return to it briefly in the discussion section. (Note that Lin and Bever [33] make the related point that subject and object relatives might both be misanalysed as non-relatives. As they put it (p. 280): “… both subject and object relative clauses can be misread as main clauses before the relativizer and the head nouns. Object relative clauses are possibly more susceptible to the main-clause misanalysis because of the initial NV sequence, following the canonical main-clause order NVN in Mandarin Chinese.” Their point also makes the storage cost based predictions difficult to uphold.).

The question now is: what does the frequency profile of RCs predict independent of the animacy issue, and at which points in the sentence does frequency affect processing? Here, one has to make a further theoretical commitment in order to derive a prediction: is the parser strictly serial or (ranked) parallel? If strictly serial, only one parse possibility would be considered at each stage; the parser would be garden-pathed (however mild this garden-path may be) in both RC types until the head noun is processed. If we assume a serial parser, it is quite difficult to determine which RC type would be preferred based on structural frequencies. If the parser is ranked parallel, then the relatively high frequency of subject relatives could facilitate processing even in the pre-head noun regions, where the most probable structure is a non-RC. Although the serial versus parallel parser issue was never really resolved in the literature [53]–[55], we follow [56], and [37], [10]–[11] in assuming a ranked parallel parser. Under this view, multiple possible structures are maintained in memory but with different ranks (determined by the probability of each parse).

Under the ranked-parallel parsing view, the higher frequency of the SR structure (compared to OR) may well play a role even before the head noun is encountered. Even though an RC structure would not be the most likely upcoming structure, such a structure would be posited with a lower rank. Under these assumptions, the cost of predicting an SR would be lower than predicting an OR from the very beginning of the sentence, although this SR advantage may well express itself in later regions due to spillover. Thus, if building an SR structure is easier than building an OR, then at the head noun (and perhaps earlier) we would expect a subject-relative advantage (the opposite to the claims of locality-based accounts).

The next necessary step is to empirically investigate the object-relative preference in the context of the Hsiao and Gibson's and Gibson and Wu's results, and to contrast DLT's integration cost predictions with the alternative frequency based explanation.

We turn next to three self-paced reading experiments that attempt to replicate the finding that object relatives are easier to process than subject relatives. All reading times were transformed (negative reciprocal) following the Box-Cox procedure; linear mixed models with varying slopes and intercepts were fit in all cases, and crossed varying intercepts were used for subject and item. Question response accuracies were analyzed using generalized linear mixed models with a binomial link function. The contrast coding in all cases was sum contrasts, with subject relatives coded as 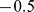 and object relatives as 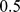. Thus, a negative sign on the coefficient represents an object relative advantage.

### Experiment 1 Results

We used Hsiao and Gibson's materials from their paper for this experiment, comparing only single embedded subject and object relatives; see 4 for examples. The methods section below provides further details.

(4) a. Single-embedded SR

**[**GAP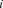 yaoqing fuhao de**]** guanyuan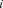 xinhuaibugui…

invite tycoon DE official have bad intentions…

‘The official who invited the tycoon has bad intentions…’

- b. Single-embedded OR

**[**fuhao yaoqing GAP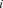 de**]** guanyuan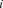 xinhuaibugui…

tycoon invite DE official have bad intentions…

‘The official who the tycoon invited has bad intentions …’

The predictions made by the integration cost metric relate to an RT comparison between SRs and ORs at the head noun and possibly the region following it. We provide analyses for the regions consisting of the relativizer, the head noun, and the word following the head noun. The dependent measure is negative reciprocal reading time, and we fitted maximal linear mixed models in the sense of Barr and colleagues [49].

#### Question-response accuracies and latencies

Subjects responded with 82.0% accuracy to all the questions. Neither the accuracies nor the question-response latencies are significantly different in the two relative clause types.

#### Analyses of reading times

**Figure 3:**
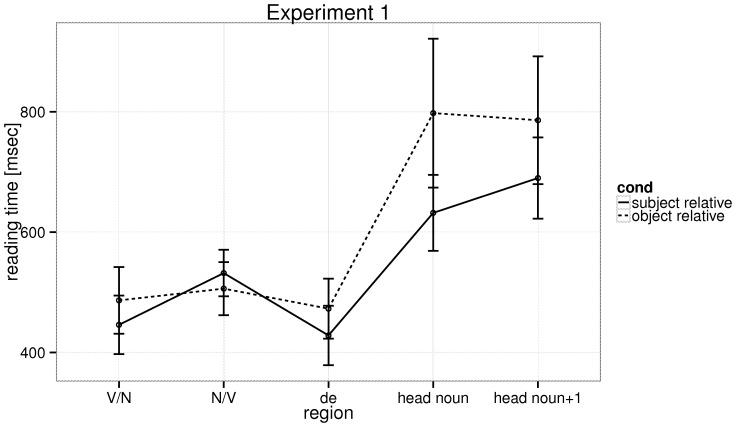
Experiment 1: The raw reading times at the five regions of interest in the relative clause types, with 95% confidence intervals.

Figure 3 shows the mean reading times for the various regions. We fit linear mixed models with relative clause type as fixed effect (coded as 0.5 for object relatives and −0.5 for subject relatives), and varying intercepts and slopes (including the varying intercept and slope correlation) for items and subjects. The Box-Cox procedure suggested a negative reciprocal transform in order to stabilize variance. We found a strong subject relative advantage at the head noun (see Table 4). This is inconsistent with the position that dependency distance is the primary determinant of Chinese relative clause processing differences. At the word following the head noun, we found no significant effect. The mean reading times at the head noun are 605 ms, SE = 37 ms (SRs) and 753 ms, SE = 47 ms (ORs); and at the word following the head noun 694 ms, SE = 34 ms (SRs) vs 800 ms, SE = 49 ms (ORs).

**Table 4:** Data Analysis: Experiment 1.

	coef.	SE	t-value
de	0.08	0.06	1.40
head noun	0.19	0.07	2.72
head noun+1	0.06	0.07	0.79

Analysis of our Experiment 1 data using negative reciprocal reading times and maximal models. A negative sign on the estimated coefficient is an object relative advantage.

In other words, this attempted replication of the claimed object-relative advantage is inconsistent with the predictions of the Dependency Locality or any other decay based account, such as the Lewis and Vasishth ACT-R based model. Rather, our results are consistent with explanations, such as the frequency-based accounts, that predict a subject-relative advantage.

Next, we repeated the above result in Experiment 2 using different stimuli and participants. Given that we found a subject-relative advantage using Hsiao and Gibson's materials, it is clearly worth investigating whether we can obtain a subject-relative advantage in a second experiment with new items.

### Experiment 2 Results

This experiment used 24 sets of stimulus items that were all single-embedded RCs; the items we derived from experiment items used in [31]. Each set had four conditions: RCs either modified the matrix-clause subject (see examples 5 above) or the matrix-clause object (we analyzed only the subject-modifying conditions in this paper). All noun phrases in the target stimuli were animate.

#### Question-response accuracies and latencies

Subjects responded with about 82% accuracy to all the comprehension questions after target items. Neither the accuracy nor question-response latencies was significantly different between responses to questions after SR items and those following OR items.

#### Analyses of reading times

**Figure 4:**
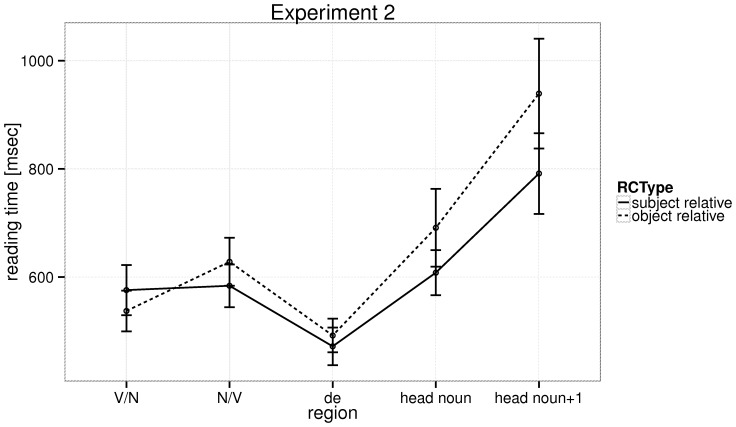
Experiment 2: The raw reading times at the five regions of interest in the relative clause types, with 95% confidence intervals.

The reading times for all regions are shown in Figure 4, and statistical analyses for the relativizer, head noun and the word following the head noun are shown in Table 5. At the head noun, we see a numerical SR advantage (coefficient: 0.07, SE = 0.06, t = 1.23), and at the word following the head noun, a strong subject-relative advantage (coefficient: 0.23, SE =  0.06, t = 4.03). The mean reading times at the head noun were 608 ms, SE = 20 ms (SRs), and 691 ms, SE = 33 ms (ORs); and at the word following the head noun 791 ms, SE = 34 ms (SRs), and 939 ms, SE = 46 ms (ORs).

**Table 5:** Data Analysis: Experiment 2.

	coef.	SE	t-value
de	0.07	0.06	1.35
head noun	0.07	0.06	1.22
head noun+1	0.23	0.06	4.03

Analysis of our Experiment 2 data using negative reciprocal reading times and maximal models. A negative sign on the estimated coefficient is an object relative advantage.

### Experiment 3

For this experiment, we obtained the items and fillers of the Gibson and Wu paper [25], and attempted a replication; recall that their experiment showed an object-relative advantage. We argued earlier that this effect is not statistically significant, but the numerical object-relative advantage they found is nevertheless potentially informative. The experiment had two conditions, subject vs object relatives, and each target sentence was preceded by a context which in principle licensed either relative clause type. This design removes, at least in principle, the temporary ambiguities that out-of-context Chinese relative clauses seem to have. See the methods section and the original Gibson and Wu paper [25] at the end for further details.

#### Question-response accuracies and latencies

Subject relatives had 89.3% correct responses, and object relatives had 87.7% correct responses; these differences, and corresponding question response latencies, did not reach significance.

#### Analyses of reading times

**Figure 5:**
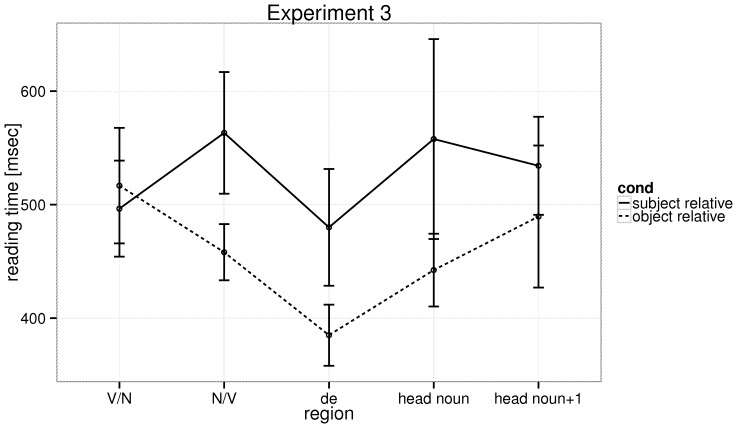
Experiment 3: The raw reading times at the five regions of interest in the relative clause types, with 95% confidence intervals.

The mean reading times for the various regions are shown in Figure 5, and statistical analyses for the relativizer, head noun, and the word following it are shown in Table 6. The Box-Cox procedure suggested a reciprocal transform.

**Table 6:** Data Analysis: Experiment 3.

	coef.	SE	t-value
de	−0.21	0.07	−2.88
head noun	−0.15	0.10	−1.44
head noun+1	−0.06	0.10	−0.59

Analysis of our Experiment 3 data using negative reciprocal reading times and maximal models. A negative sign on the estimated coefficient is an object relative advantage.

At the relativizer, we see an object-relative advantage; this is inconsistent with the predictions of the frequency-based accounts but it is also inconsistent with the predictions of the storage and integration accounts developed in [20], [25] for Chinese. At the head noun, which is the critical region, a marginal effect of relative clause type was seen, with a numerical object-relative advantage (for the negative reciprocal transform, coefficient: −0.15, SE = 0.10, t = −1.44; cf. the estimates in Gibson and Wu: coefficient: −0.0762, SE = 0.09, t = −0.85). This is numerically consistent with the claim of Gibson and colleagues that an object-relative advantage exists in Chinese. At the word following the head noun, no effect was seen (coefficient: −0.0562, SE = 0.10, t = −0.6). The mean reading times at the head noun were 558 ms, SE = 43 ms (SRs), and 442 ms, SE = 17 ms (ORs), and at the word following the head noun 534 ms, SE = 23 ms (SRs), and 489 ms, SE = 30 ms (ORs).

**Figure 6:**
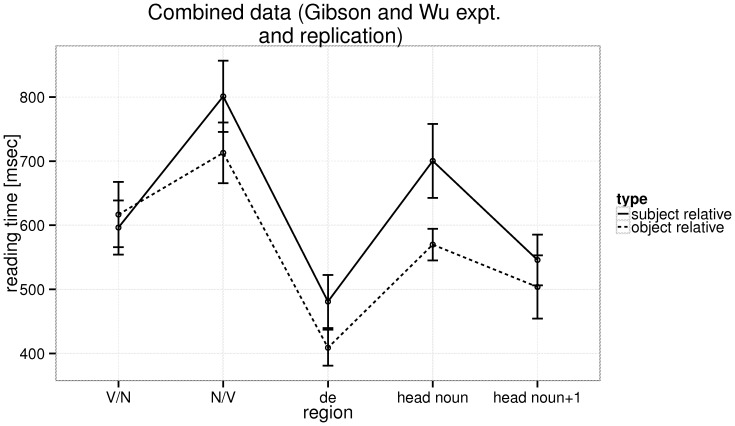
Experiment 3 and the original Gibson and Wu dataset combined: The raw reading times at the five regions of interest in the relative clause types, with 95% confidence intervals.

We also combined the original dataset of Gibson and Wu with our own replication (yielding a total of 77 participants); the dependent measure was negative reciprocal reading time. Figure 6 shows the raw reading times with 95% confidence intervals for all the relevant regions of interest; Table 7 shows the results of the statistical analyses for the relativizer, head noun, and the word following it. We see a statistically significant object relative advantage at the relativizer, and the head noun. No effect due to integration cost or storage cost is predicted by the Dependency Locality Theory at the relativizer, because context is supposed to make it unambiguous that a relative clause is being processed. Thus, it is possible that the object relative advantage seen at the head noun could be spillover from the preceding region; this in turn implies that, at least in this dataset, the subject relative may be more difficult to process than the object relative due to reasons that have nothing to do with integration or storage costs. One explanation, suggested by C. Lin [38], strikes us as plausible: he shows that the object relative advantage is only seen when the preceding context has the same thematic ordering as the target sentence. This priming explanation would explain the early object relative advantage we see in the above analysis: the participant would have just read a context ending with “B verbed another A”, and then they read the object relative, which has the pattern “B verbed relativizer A”; this could be easier than the subject relative “verbed B relativizer A”. Note however that Lin does not really demonstrate that there is an interaction (no statistics are provided in the paper regarding the interaction); he only showed that the object relative advantage is seen when a priming configuration exists. Thus, his proposal needs further investigation. Together, the Gibson and Wu design and Lin's contribution to the issue have the potential to clarify the question. But our experiment 3 data, a replication of Gibson and Wu's original study, cannot be explained by storage or integration cost metrics.

**Table 7:** Data Analysis: Experiment 3 and Gibson and Wu data combined.

	coef	SE	t-value
de	−0.19	0.05	−3.45
head noun	−0.10	0.05	−2.15
head noun+1	−0.01	0.05	−0.16

The original Gibson and Wu data and Experiment 3 combined: The raw reading times at the five regions of interest in the relative clause types, with 95% confidence intervals.

## Discussion

We investigated the processing of subject and object relatives in Chinese, and demonstrated that two of the three experiments we carried out are inconsistent with the claim that Chinese relative-clause processing difficulty can be explained by dependency distance. Distance-based accounts predict increased difficulty in SRs at the head noun: in the Dependency Locality Theory [29], this is because, compared to the OR, one extra discourse referent intervenes in the SR between the head NP and the gap it is coindexed with; in the decay and interference-based account [57], the SR should be more difficult than the OR because the gap site is more distant in the SR than OR case and has decayed more and in addition suffers from retroactive interference due to the intervening object in the SR. In experiments 1 and 2, at the head noun and/or the region following it, SRs are processed faster than ORs, which goes against integration cost and decay and interference accounts. Experiment 3, the replication of Gibson and Wu's study, does show evidence for an object-relative advantage, but these results cannot be explained by any distance-based account, because the OR advantage begins at the relativizer, where no effect is predicted by either storage or integration accounts. The storage account in the DLT would predict no difference at the relativizer since the same number of heads will be predicted at this point; the integration account's predictions only come into play at the head noun. Thus, overall, the data from these three experiments speak against a storage and distance-based account.

Why are we unable to obtain the same results as Gibson and colleagues in experiments 1 and 2? Apart from our earlier observations about their two experiments, which we think could have introduced sources of bias in their analyses and data, a fact that seems to have gone unnoticed in the literature is that the original Hsiao and Gibson study [20] in fact found a *subject* relative advantage at the head noun in single embeddings (just as we did in Experiment 1), but their result did not reach statistical significance. The OR advantage was seen only in double-embedding items, and there the effect was found either at non-identical regions (noun versus verb), or at the second *de*, which was preceded by different words (noun or verb), which may lead to spillover effects at *de* (a claim that can be tested by reanalyzing Hsiao and Gibson's data). [25] found an object relative advantage at the head noun, but this effect is driven by a few data points, as we showed earlier. Related to this, the statistical model's assumptions are not satisfied in the original Gibson and Wu analysis; the residuals are far from normally distributed. Once model assumptions are satisfied, we see no effect in their data at the head noun. Taking these facts into consideration, our effects do not seem entirely inconsistent with previous work.

### Synthesizing the evidence: A bayesian meta-analysis

Nevertheless, a skeptical reader would be completely justified in concluding that our own studies' results may simply be a consequence of random variability (or bias on our part). Indeed, we have encountered reviewer comments to the effect that the published results show effects of both types (subject- or object-relative advantage), and the present work does not clarify the issue at all.

But it is true that the previous work and ours provides no information at all about the facts about Chinese? There is a quantitative answer to that question. We can ask ourselves: what should the researcher believe given the data? That is, what is the probability of there being a subject-relative advantage given the data? A bayesian meta-analysis that synthesizes our knowledge about Chinese relative clauses would allow us to formalize our prior belief, and, more importantly, the extent to which our belief should shift given new data.

Bayesian random effects meta analysis a well-known methodology in medicine and other areas and is used primarily for evidence synthesis [58]. An important advantage of using the bayesian approach is that we can draw probabilistic inferences from the posterior distribution, which allows us to calculate, given the data, the probability of the coefficient being negative or positive, i.e., the probability that Chinese has a subject or object relative advantage. In other words, we can explicitly talk about our beliefs in terms of probabilities.

We describe the random effects model next. Let 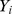 be the effect size in the 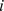-th study, where 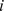 ranges from 1 to k (in this study, k = 15). The unit is milliseconds; a positive sign means a subject relative advantage and a negative sign an object relative advantage. Let 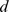 be the underlying effect size, to be estimated by the model. Let 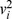 be the estimated within-study variance.

Then, our model is:
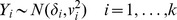
(1)where
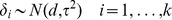
(2)


The variance parameter 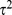 represents between-study variance and precision is represented by 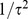. The prior for 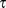 could be a uniform distribution: 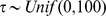, or a gamma distribution: 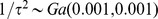. The choice of upper bound for the uniform distribution is based on experience with psycholinguistic studies.


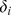
 are assumed exchangeable: they are different, but a priori we cannot predict the differences in their magnitudes. 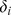 are random samples from a distribution of effect sizes; this is why the model is called a random effects model.

Plausible values of the subject/object relative clause advantage can be assumed to range between −300 and 300 ms. But we will assume three different levels of uncertainty: The 95% range is (a) 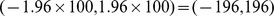; (b) 
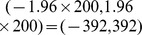
; and (c) 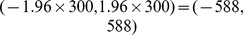.

We therefore use three priors for 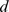: 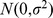, with 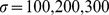. These priors correspond to an agnostic starting point with increasing levels of uncertainty about the range of plausible values for the relative clause processing difference. The model was fit using JAGS version 3.1 [59] and R [60]. The initial values were 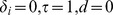 and 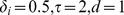; we checked that the results do not depend on the initial values. We ran two chains with a burn-in of 5000, and the MCMC sampling had 10,000 runs, with thinning 20. Convergence was checked visually and using the Gelman and Rubin diagnostic [61]; the chains converged in all our models.

All available data were used for the analysis, including those from the present study. The modeling results are summarized in Table 8.

**Table 8:** Results of meta-analysis.

Prior on 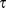 or 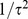	Prior on 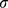 for 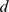	Posterior distrn.	Posterior prob.
		(median, 95% CrI)	SR advantage (%)
Unif(1,100)	100	17 (−27, 59)	80
Unif(1,100)	200	18 (−23, 56)	81
Unif(1,100)	300	19 (−22, 60)	79
Ga(0.001,0.001)	100	17 (−31, 63)	78
Ga(0.001,0.001)	200	16 (−30, 65)	79
Ga(0.001,0.001)	300	17 (−32, 61)	79

The posterior distribution of the coefficient using different priors; the final column displays the posterior probability that the coefficient is positive, i.e., the probability that Chinese has a subject relative advantage. See text for details.

The central insight from this analysis is that the data do not lead us to believe strongly in the object relative advantage; indeed, given the data, the probability of Chinese having a subject-relative advantage is approximately 78–80%. In other words, if one believes that there is any difference at all between subject and object relative clause processing times at the head noun in Chinese, given the data one's belief should be in favor of a subject-relative advantage. One reaction to this analysis might be that a summary of the means in Table 1 gives us information not very different from the bayesian analysis. This misses the point that we can assign a posterior probability to the subject-relative advantage given the data, which in turn allows us to quantify our uncertainty about our beliefs. Making a probabilistic statement given what we know so far is much more useful than impressionistically talking about what the evidence tells us.

In sum, given the data that are currently available to investigate this issue, although it is certainly not as clear as in comparable sentences for English, the evidence weighs in favor of the subject-relative advantage. Importantly, it is not the case that the evidence available so far regarding Chinese relative clause processing is completely unclear.

### Alternative explanations for the subject-relative advantage

We discuss here some possible explanations for the SR advantage in Chinese. The evidence from experiments 1 and 2 is consistent with frequency based explanations: the subject-relative occurs more frequently, and therefore it is easier to access that structure when a choice point is reached. This idea can be formalized within the expectation-based account of Levy [62] and Hale [22]. Of course, one can legitimately criticize such a frequency-based account as being descriptive and not explanatory because such an account cannot explain *why* subject relatives are more frequent than object relatives—surely the answer to that question would be the real explanation, not frequency, which is merely a side-effect of the preference to produce subject relatives. Thus, the frequency account is not really an explanation, but rather begs the question: why do subject relatives occur more often? As a reviewer (Roger Levy) points out, one reason that subject relatives may be more frequent is that, given that relative clauses serve to fix reference, it may be easier to fix reference by mentioning the action that an (animate) entity does than mentioning an action that is done to an entity. A related possibility is that subjects may simply be more accessible than objects [63]. For experiment 3, we do see evidence for an object-relative advantage, but as C. Lin has argued, this may be due to a priming effect arising from the thematic roles from the immediately context priming the processing of the target sentence; if this is correct, the context in experiment 3 does not help in resolving the issue. Overall, our data show no evidence at all for distance-based explanations of Chinese relative clause processing.

Although our data do call into question dependency distance-based explanations of relative clause processing in Chinese, we do not claim that dependency distance plays no role in language processing in general or even in relative clause processing in general. Previous work on various languages with SVO order has shown clear evidence for locality effects (e.g., [47], [64], [66], [67]); we even find locality effects in head-final languages like Hindi [65], which have been argued to be show anti-locality effects. Levy [62] has pointed out that in general a two-factor model, including both expectation and locality-based effects, is necessary to explain a range of effects. There are many important facts about dependency resolution that can be explained by locality accounts and not by frequency based accounts, and it is clear that both operate in tandem [68], [69]. For Chinese relative clauses, this could mean that although locality effects might be coming into play exactly as Gibson and colleagues suggest, expectation effects dominate and mask the object-relative advantage. In languages like English, both expectation and locality based effects predict a subject relative advantage, making it difficult to determine what the underlying cause for the preference is (the fact that both factors yield a subject-relative advantage may be the reason that the subject vs object relative clause difference is stronger in English). Chinese may eventually help to clarify the situation.

A final point about the role of working memory accounts in Chinese relative clause processing: Gibson and Wu [25] have argued that the conclusion in [70] that Chinese object relatives are easier to process than subject relatives in aphasics might support the working memory account of Chinese relatives. As they put it:

In contrast, it has recently been observed that there is a reverse pattern of difficulty in Chinese: some Chinese aphasic patients cannot reliably answer comprehension questions about SRCs, although they perform well on ORCs [70]. These results from the neuropsychological studies are strikingly similar to the current literature on RC processing in healthy populations: English speakers have more difficulty with ORCs than SRCs, whereas Chinese speakers have more difficulty with SRCs than ORCs. The patient data therefore further strengthen the conclusion reached in the current study: that a memory component is essential in order to explain the existing patterns of linguistic behavior.

It is true that some aphasic patients cannot reliably answer comprehension questions about SRCs. But the number of such patients is small: only two out of the six patients examined by Su and colleagues in one of two versions of their experiments showed this pattern (see Table 7 in [70]). In the six patients, the difference between subject and object relative error rates was 31, 25,−3,−5,−4, and −5%. It is difficult to see this as a strong argument in favor of working memory accounts of Chinese relative clause processing. Note that Su and colleagues were comparing working memory accounts and the trace deletion hypothesis (TDH) [71] and argue in favor of the TDH. We are not arguing in favor of the trace deletion hypothesis as an explanation for Chinese relative clause processing. In fact, in other work [72] we provide support from computational modeling for working memory explanations for sentence comprehension deficits in aphasia (our notion of working memory there is different from the Dependency Locality Theory's). Our point here is merely that the data from Chinese aphasic patients currently provides little credible evidence in favor of the working memory account. However, had the Chinese evidence shown that most or all aphasics have more difficulty with subject than object relatives, then this could provide important evidence for the working memory explanation, provided other explanations such as the TDH can be eliminated. More research on Chinese aphasics seems to be needed to address these issues.

### A note on replicability in psycholinguistics

Finally, it should not be forgotten that our work is also open to challenge. For example, it is quite possible that the local ambiguities inherent in the sentences in experiments 1 and 2 are confounding the true underlying pattern (although we doubt it: see [73], [74] for evidence for a subject relative advantage even when the local ambiguities in Chinese relatives are eliminated). In order to clarify such questions, more replications of published work are needed. This is not happening because replication attempts are currently not considered important in experimental research in psycholinguistics; this is clear from the recent increase in discussion on the topic [75], at least in psychology. Statistical significance has little value per se if a result is not replicable; and a replication (or a failed replication) is just as newsworthy as an original finding. We are optimistic that this fact about statistical inference will eventually become a part of the culture in psycholinguistics, and replication attempts will come to be valued.

## Materials and Methods

All R code and data, along with the stimulus items used for the experiments, are available from the first author.

### Ethics statement

The Ethics Commission of the University of Potsdam has provided written approval of this project (Proposal number 32/2012). A document certifying this is attached. Subjects did not have to sign an informed consent form because the data were collected in an anonymized manner.

### Experiment 1

#### Participants

60 university students in Taiwan were paid 200 TWD for taking part in this self-paced reading experiment. The average age was 20 and all participants were native speakers of Mandarin Chinese using traditional characters.

#### Stimuli and fillers

Experiment 1 used twenty sets of single-embedded RC items published in [20]. All noun phrases in the target stimuli were animate. RCs were sentence-initial and modified subjects of the matrix clause. Single-embedded relative clauses in example (1) are repeated below as (5). We only show the first five words of this item sentence. Each sentence in Experiment 1 continued beyond the fifth word. The fifth word in (5), the one following the head noun, is the main verb, *xinhuaibugui* ‘have bad intentions’. However, a predicate-modifying adverb (i.e. *hen* ‘very’) could also appear at this position in other stimulus items. This arrangement prevented the sentence wrap-up effect from occurring in the verb.

(5) a. Single-embedded SR

**[**GAP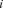 yaoqing fuhao de**]** guanyuan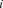 xinhuaibugui…

invite tycoon DE official have bad intentions…

‘The official who invited the tycoon has bad intentions…’

- b. Single-embedded OR

**[**fuhao yaoqing GAP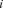 de**]** guanyuan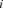 xinhuaibugui…

tycoon invite DE official have bad intentions…

‘The official who the tycoon invited has bad intentions …’

In addition to the stimulus items, 50 fillers with varying syntactic structures were randomly interspersed between items, with the constraint that at least one filler intervened between two items. Both items and fillers were presented in traditional Chinese characters.

#### Procedure

The experiment used the non-cumulative self-paced moving window method [36] used standardly in psycholinguistics and in the previous work on Chinese relative clauses [20], [25]. We presented stimulus items using Douglas Rohde's Linger software (http://tedlab.mit.edu/ 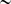
dr/Linger/); this was also the software used in [20], [25]. First, participants were explained the task and several practice sentences were presented to familiarize them with the presentation format. Their task was to press the space bar in order to view each successive segment. At the beginning of each trial, the participant saw a mask of hyphens preserving the line-breaks and inter-word spaces of the upcoming sentence; each time the space bar was pressed, a new segment was unmasked while previous and following segments were kept masked, until the participant had read the whole sentence. RTs (in milliseconds) were taken as a measure of relative momentary processing difficulty. In all the trials, a simple yes/no comprehension question followed the sentence to ensure that participants were reading for comprehension. The segmentation of the sentences into phrases was as demarcated by white spaces between words in (5). Compound nouns *sijia zhentan* “private detective” and *xiju yanyuan* “comedy actor” (in items 11 and 12 of [20]) were each presented as two segments in the experiment. However, their reading times were treated as a whole in the analysis.

In all experiments, a linear mixed model (the **lmer** function in R) [76] was fit, with participants and items as crossed random effects, and the SR/OR asymmetry as the fixed effect, coded as an orthogonal centered sum contrast (SRs were coded −0.5, and ORs as 0.5). We always fit varying intercepts and slopes models and a correlation parameter.

### Experiment 2

#### Participants

61 college students in Dalian, China with an average age of 20 were each paid 15 RMB for taking part in Experiment 2. All participants were native speakers of Mandarin Chinese using simplified characters.

#### Stimuli and fillers

Experiment 2 had a 2
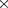
2 factorial design; in this paper we analyze only one factor from this design because the other factor was not relevant to the paper. 24 sets of stimulus items were all single-embedded RCs revised from experiment items used in Lin and Bever 2007. Each set had four conditions: RCs either modified the matrix-clause subject (see examples 5 above) or the matrix-clause object (we analyzed only conditions a,b in this paper). All noun phrases in the target stimuli were animate. We split the target sentences into four lists in a Latin-Square design. Each list was combined with 80 filler sentences. All materials were presented to participants in simplified characters.

#### Procedure

Experiment 2 had the same procedure as Experiment 1. The Linger software recorded RTs at each word of a presented target sentence as well as the answer to a following yes/no comprehension question.

### Experiment 3

#### Participants, stimuli, and procedure

40 college students in Dalian, China were each paid 15 RMB for taking part in Experiment 2. All participants were native speakers of Mandarin Chinese using simplified characters. Experiment 3 was a replication of [25]; that paper should be consulted for further details. The same procedure was used as in as Experiment 1.
